# Supplementary material for: Multidimensional helical dichroism from a chiral molecular nanoassembly
Source: Nat Commun. 2026 Jan 29;17:1829. doi: 10.1038/s41467-026-68540-y (PMC12920910; doi:10.1038/s41467-026-68540-y)
Supplement: Supplementary file 1 — Supplementary Information [file 41467_2026_68540_MOESM1_ESM.pdf]

Supporting Information for

Multidimensional helical dichroism from a chiral molecular nanoassembly

Yusheng Jin[1], Xinghao Wang[1], Zhijie Xia[1], Xiaoxu Rao[1], Xiaomei Chen[2], Kaixuan Li[2], Yucheng Jiang[2,3], Jiaru Chu[1], Dong Wu[1]*, Cheng-Wei Qiu[2,4,5]*, Jincheng Ni[1,6]*

[1]*CAS Key Laboratory of Mechanical Behavior and Design of Materials, Department of Precision Machinery and Precision Instrumentation, University of Science and Technology of China, Hefei 230027, China.*

[2]*Department of Electrical and Computer Engineering, National University of Singapore, Singapore 117583, Singapore.*

[3]*Jiangsu Key Laboratory of Intelligent Optoelectronic Devices and Chips, School of Physical Science and Technology, Suzhou University of Science and Technology, Suzhou, 215009, China.*

[4]*National University of Singapore Suzhou Research Institute, No. 377 Linquan Street, Suzhou, Jiangsu, China*

[5]*Department of Physics, National University of Singapore, Singapore, Singapore*

[6]*State Key Laboratory of Opto-Electronic Information Acquisition and Protection Technology, Anhui University, Hefei, 230601, Anhui, P. R. China.*

**To whom correspondence should be addressed: dongwu@ustc.edu.cn; chengwei.qiu@nus.edu.sg; njc@ustc.edu.cn.*

Supplementary Note 1: SAM to OAM conversion

The non-local nature of circularly polarized light normally requires a considerable number of chiral nanoassemblies for effective CD measurements. Moreover, the tight focusing of circularly polarized light can lead to the photonic SAM to OAM conversion[1], hindering the local interaction with single nanoscale structures.

Circularly polarized light rotates its electric field along a circular path at a constant angular velocity in a plane perpendicular to the direction of propagation. This rotation is uniform and non-local, making circularly polarized light effectively a periodic optical field with arbitrary period. Due to this property, chiral microstructures can be arranged in a periodic manner to enhance the CD signal. Meanwhile, when circularly polarized light is focused by a high numerical aperture (NA) lens[2,3], SAM is partially converted into OAM due to spin-orbit interaction of light[4–7]. For nonparaxial circularly polarized beams with wavevectors forming a cone at an angle θ, SAM and OAM can be defined as:

$$\mathbf{S} = \sigma \cos\theta \frac{\boldsymbol{P}}{P} \tag{S1}$$

$$\mathbf{L} = [l + \sigma(1 - \cos\theta)] \frac{\boldsymbol{P}}{P} \tag{S2}$$

Where $\mathbf{S}$ is the SAM, $\mathbf{L}$ is the OAM.

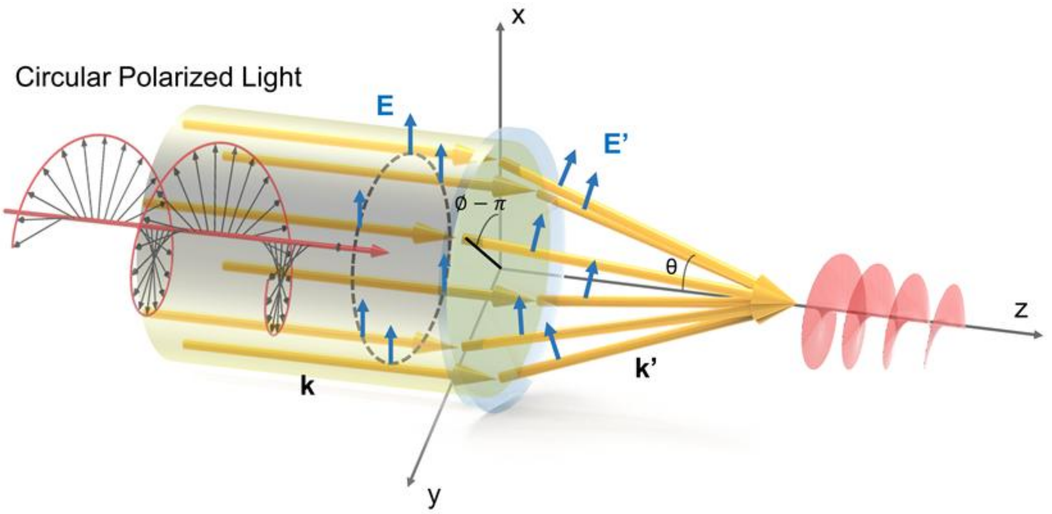

Fig. S1. Focusing of circular polarized vortex light by a spherical high-NA lens.

In the following, we will break down the derivation of this conclusion into three distinct sections: 1) transformation relations of the electric field before and after incidence; 2) separation of SAM and OAM components of the electric field; 3) the incidence of circularly polarized light with OAM.

(1) Transformation relations of the electric field before and after incidence

According to the adiabatic approximation that does not consider the polarization dependence of the refraction coefficients, the Debye–Wolf theory assumes that partial waves do not change their polarization state in the local coordinate system attached to the ray. For the electric field deflected at an angle $\emptyset$, its local coordinate system first rotates counterclockwise by $\emptyset$ around the z-axis, then rotates by θ around the y-axis. Let the rotation matrix for a rotation around the axis by an angle θ be denoted as $R_i(\theta), i = x, y, z$,then we have

$$\boldsymbol{E_0'} = R_z(\emptyset)R_y(-\theta)R_z(-\emptyset)\boldsymbol{E_0} \qquad (S3)$$

where $\boldsymbol{E_0}$ is the direction vector of the incident electric field, $\boldsymbol{E_0'}$ is the direction vector of refracted electric field, $R_y(-\theta)$ is the transformation matrix representing the refraction in the local coordinate system and $R_z(-\emptyset)$ denotes the transformation matrix that maps the basis vectors of the global coordinate system to those of the local coordinate system. Then

$$\boldsymbol{E_0'} = \begin{pmatrix} \cos^2\emptyset\cos\theta + \sin^2\emptyset & sin\,\emptyset\cos\emptyset\cos\theta - sin\,\emptyset\,cos\emptyset & sin\theta cos\emptyset \\ sin\,\emptyset\cos\emptyset\cos\theta - sin\,\emptyset\,cos\emptyset & \sin^2\emptyset\cos\theta + \cos^2\emptyset & sin\emptyset sin\theta \\ -sin\theta cos\emptyset & -sin\theta sin\emptyset & cos\theta \end{pmatrix}\boldsymbol{E_0} \qquad (S4)$$

For convenience, we rewrite the equation in the circular polarization basis:

$$\boldsymbol{E_0'} = V^{-1}R_z(\emptyset)R_y(-\theta)R_z(-\emptyset)V\boldsymbol{E_0}, V = \frac{1}{\sqrt{2}}\begin{pmatrix} 1 & 1 & 0 \\ i & -i & 0 \\ 0 & 0 & \sqrt{2} \end{pmatrix} \qquad (S5)$$

where V is the transformation matrix between the circular polarization basis and the Cartesian orthogonal basis. Therefore,

$$\boldsymbol{E_0'} = \begin{pmatrix} a & -be^{-2i\emptyset} & \sqrt{2ab}e^{-i\emptyset} \\ -be^{2i\emptyset} & a & \sqrt{2ab}e^{i\emptyset} \\ -\sqrt{2ab}e^{-i\emptyset} & -\sqrt{2ab}e^{-i\emptyset} & a-b \end{pmatrix}\boldsymbol{E_0} \qquad (S6)$$

where $a = cos^2\left(\frac{\theta}{2}\right), b = sin^2\left(\frac{\theta}{2}\right)$.

Considering energy conservation, we have

$$\boldsymbol{E}' = \sqrt{\cos\theta}\begin{pmatrix} a & -be^{-2i\emptyset} & \sqrt{2ab}e^{-i\emptyset} \\ -be^{2i\emptyset} & a & \sqrt{2ab}e^{i\emptyset} \\ -\sqrt{2ab}e^{-i\emptyset} & -\sqrt{2ab}e^{-i\emptyset} & a-b \end{pmatrix}\boldsymbol{E} \qquad (S7)$$

Here, $\boldsymbol{E}$ is the incident electric field, $\boldsymbol{E}'$ is the refracted electric field.

(2) Separation of SAM and OAM components of the electric field

First, we provide the SAM and OAM operators for the z-component in the Cartesian coordinate system

$$L_z = -i\frac{\partial}{\partial\emptyset}, (S_z)_{ij} = -\varepsilon_{zij} \qquad (S8)$$

where $\emptyset$ is the azimuthal angle (either in coordinate or momentum space, depending on representation) and ε_{zij} is the Levi-Civita symbol.

For convenience, we rewrite the equation in the circular polarization basis:

$$L_z = -i\frac{\partial}{\partial\emptyset}, \sigma = diag(1,-1,0) \qquad (S9)$$

For a given electric field $\boldsymbol{E}$, the OAM and SAM densities can be calculated as follows:

$$l_z = -i\frac{\boldsymbol{E}^*\cdot\partial_{\emptyset}\boldsymbol{E}}{\boldsymbol{E}^*\cdot\boldsymbol{E}}, s_z = \frac{\boldsymbol{E}^*\cdot\sigma\boldsymbol{E}}{\boldsymbol{E}^*\cdot\boldsymbol{E}} \qquad (S10)$$

(3) Circularly-polarized vortex beam incidence

The electric field of the incident circularly-polarized vortex beam can be written as:

$$E_l^\sigma \approx e^\sigma E_l(\theta,\emptyset), E_l(\theta,\emptyset) = F_{|l|}(\theta)e^{il\emptyset} \qquad (S11)$$

and $F_{|l|}(\theta) \propto sin^{|l|}\theta$ for the vortex beams with the waist much larger than the entrance pupil. From Eq. (S5) the refracted field is:

$$\boldsymbol{E}' = \sqrt{\cos\theta}\, e^\sigma F_{|l|}(\theta)e^{il\emptyset}\begin{pmatrix} a & -be^{-2i\emptyset} & \sqrt{2ab}e^{-i\emptyset} \\ -be^{2i\emptyset} & a & \sqrt{2ab}e^{i\emptyset} \\ -\sqrt{2ab}e^{-i\emptyset} & -\sqrt{2ab}e^{-i\emptyset} & a-b \end{pmatrix} \qquad (S12)$$

If $\sigma = +$,

$$\boldsymbol{E}'_{+} = \sqrt{cos\theta}F_{|l|}(\theta)\begin{pmatrix} cos^2\frac{\theta}{2}e^{il\phi} \\ -sin^2\frac{\theta}{2}e^{i(l+2)\phi} \\ -\sqrt{2}sin\frac{\theta}{2}cos\frac{\theta}{2}e^{i(l+1)\phi} \end{pmatrix} \quad (S13)$$

If $\sigma = -$,

$$\boldsymbol{E}'_{-} = \sqrt{cos\theta}F_{|l|}(\theta)\begin{pmatrix} -sin^2\frac{\theta}{2}e^{i(l-2)\phi} \\ cos^2\frac{\theta}{2}e^{il\phi} \\ -\sqrt{2}sin\frac{\theta}{2}cos\frac{\theta}{2}e^{i(l-1)\phi} \end{pmatrix} \quad (S14)$$

Eq. (S8) brings about

$$s_z = \sigma\cos\theta \quad (S15)$$

$$l_z = [l + \sigma(1 - \cos\theta)] \quad (S16)$$

When the lens diameter is much larger than its focal length, most regions on the incident surface refract at angles close to 90 degrees relative to the optical axis, leading to almost complete conversion of circularly polarized light into the vortex light.

Therefore, for both Gaussian beams (l=0) and OAM beams, a portion of the SAM is converted into OAM due to the presence of a significant longitudinal field component with a NA=0.9 in our experiments.

Supplementary Note 2: Experimental characterization of vortex beams

The optical field distributions of vortex beams at the focal plane, captured by a charge-coupled device (CCD) camera, exhibit a characteristic donut-shaped intensity profile (Fig. S2b). Vortex beams with opposite OAM modes theoretically exhibit identical intensity patterns but feature opposite phase gradients along the azimuthal direction in the transverse plane. The donut-shaped profile's diameter d is determined by measuring the distance between the two highest intensity points in the cross-sectional intensity distribution. Experimental results, as shown in Fig. S2a, reveal that the diameter d of the focused vortex beam grows linearly with the topological charge. This behavior is in agreement with the expected dependent scaling relationship under focusing conditions:

$$d \propto \frac{2\lambda}{NA}\left(1 + \frac{|l|}{l_0}\right) \qquad (S17)$$

where λ is the wavelength of light, NA is the numerical aperture of objective lens and l_0 is the constant. We also measure the three-dimensional intensity distribution of vortex beam with topological charge ℓ=+30 (Fig. S3).

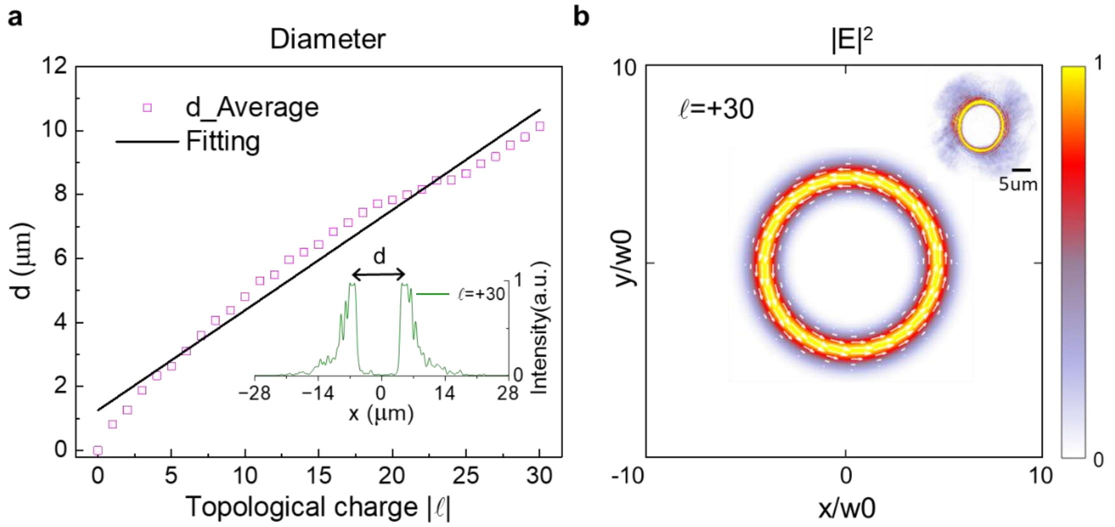

Fig. S2. Two-dimensional characterization of the vortex beams used in the experiment. a, Measured diameter d of the vortex beam as a function of topological charge. The results presented here are the average of the positive and negative topological charge cases for each topological charge value ℓ. (Inset) Intensity distribution along the central axis of the donut-shaped profile. The distance between the

two intensity peaks represents the diameter of the vortex beam. The solid line represents the linear fitting of measured diameter d. **b**, The simulated optical field distribution of the incident vortex beams with topological charge $\ell = +30$. The length and orientation of the white arrow are indicative of the magnitude and direction of the momentum density in the x-y plane, respectively. (Inset) The corresponding experimental optical field distribution.

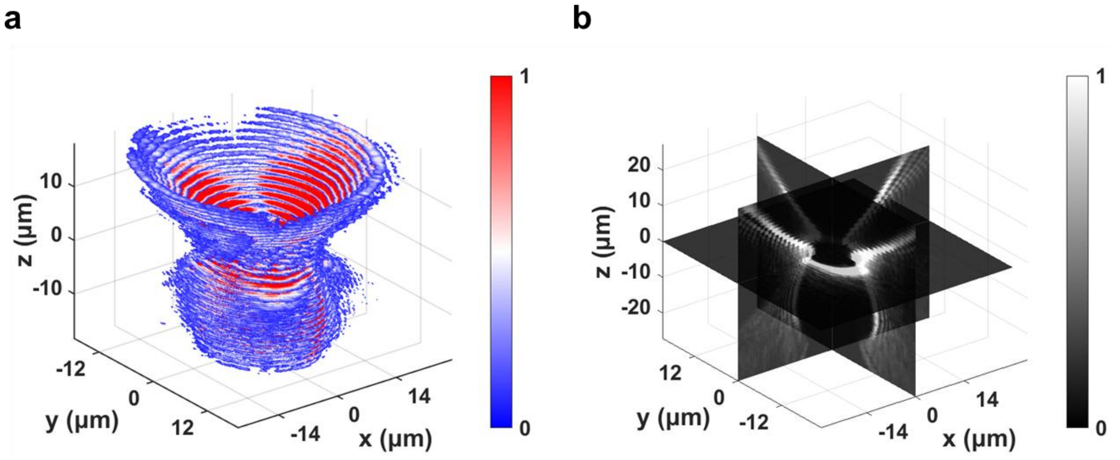

Fig. S3. Experimental three-dimensional intensity distribution of vortex beam with topological charge $\ell = +30$. a, Experimental three-dimensional intensity distribution. **b**, Slices from three cross-sections.

Supplementary Note 3: Simulation of angular momentum flux densities without structures

Simulations of angular momentum flux densities without structures are conducted to better understand the differences between using circularly polarized light and vortex beams for the chiroptical detection of microstructures (Fig. S4). To ensure consistent total angular momentum of the incident photons, circularly polarized light with $\sigma = +1$ and vortex light with topological charge $\ell = +1$ are used in the simulation, both with the same power. As expected, in the absence of a structure, the angular momentum flux density of circularly polarized light is primarily its spin component, while that of vortex light is primarily its orbital component. The higher local angular momentum flux density for vortex beams, despite equal total angular momentum flux, suggests an advantage using vortex light for chiroptical detection.

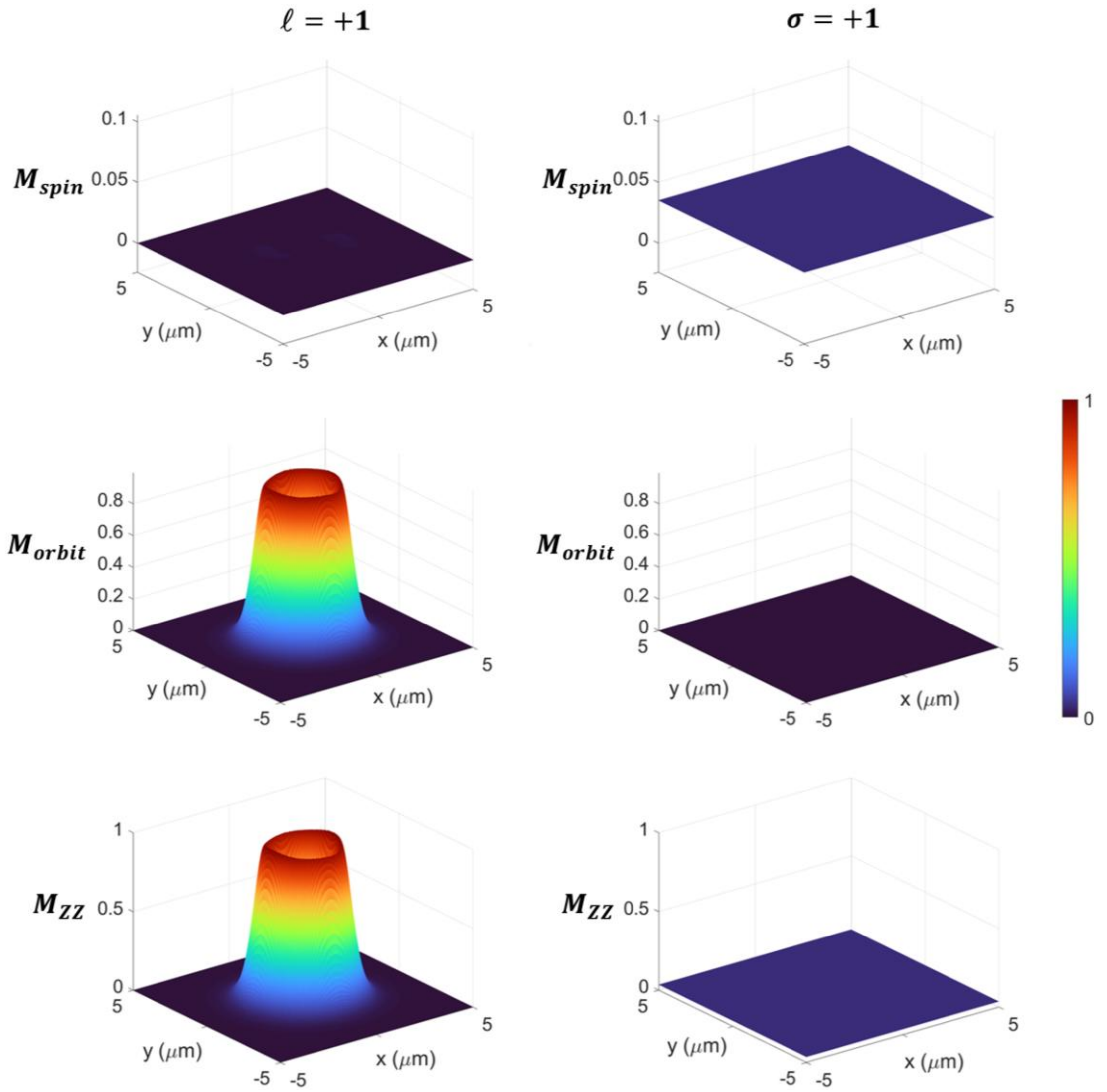

Fig. S4. Local angular momentum flux densities for circularly polarized light and vortex beam without structures. The angular momentum flux density of circularly polarized light primarily manifests as a spin component M_{spin}, while that of vortex light primarily manifests as an orbital component M_{orbit}.

Supplementary Note 4: Selection of refractive index

Although most amino acids, including cystine crystals, have a refractive index of 1.6, previous studies have confirmed that cystine crystal structures synthesized by this method can be transformed into CdS nanoparticles at room temperature[8]. Consequently, the refractive index of chiral nanoassemblies is set to 2.5. To provide a comprehensive understanding, we have also compared the simulation results using refractive indices of 1.6 (representative of native cystine crystals) and 2.5 (representative of CdS nanoparticles). This comparison allows us to evaluate the impact of the material transformation on the optical behavior, particularly in the context of chiroptical detection and interactions with OAM beams (Fig. S5). The use of refractive index 2.5 in our primary simulations ensures that our theoretical model aligns with the realistic optical properties of the transformed chiral nanoassemblies under the studied conditions.

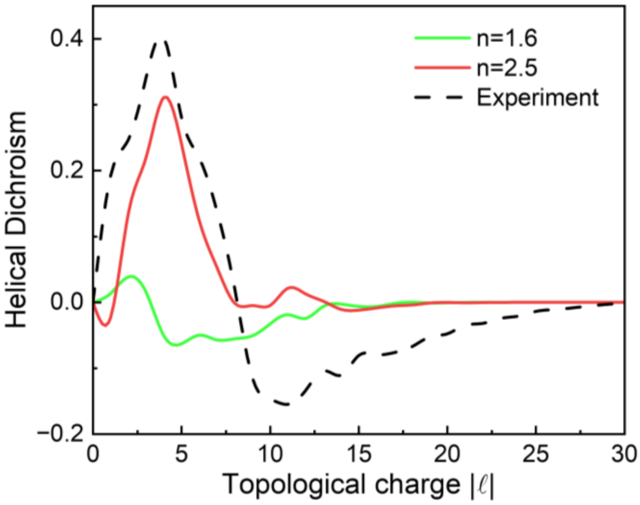

Fig. S5. Comparison of simulated HD signal for refractive indices n = 1.6 and n = 2.5. The dashed lines correspond to the experimental data.

Supplementary Note 5: Simulation of intensity distributions on chiral nanoassemblies

We present the simulated intensity distributions of vortex beams with topological charge $\ell = \pm 4$ after interaction with the D-Cys structure (Fig. S6). Electromagnetic field simulations provide a direct visualization of the physical principles governing chiroptical HD: vortex beams with opposite topological charges generate clearly distinguishable optical intensity patterns. Stronger light-matter coupling in the D-Cys structure is observed with vortex beams possessing a topological charge of ℓ = -4, as evidenced by the higher internal electric field intensity compared to beams with ℓ = +4. HD response in the transmission mode is also simulated (Fig. S7). The transmission mode HD signal exhibits an opposite sign but follows a similar trend to the reflection mode.

To clarify the origin of HD, we have conducted a multipolar decomposition analysis of the D-cystine-based chiral nanoassemblies (Fig. S8), following established methodologies[9]. The results demonstrate that the electric quadrupole (EQ) component contributes significantly to the electromagnetic field response under incident vortex light with OAM states ℓ = +4 and ℓ = −4. This significant EQ contribution accounts for the strong HD response observed, despite the nanoassemblies lying flat on the substrate, as the higher-order multipole interactions enhance the chiroptical response.

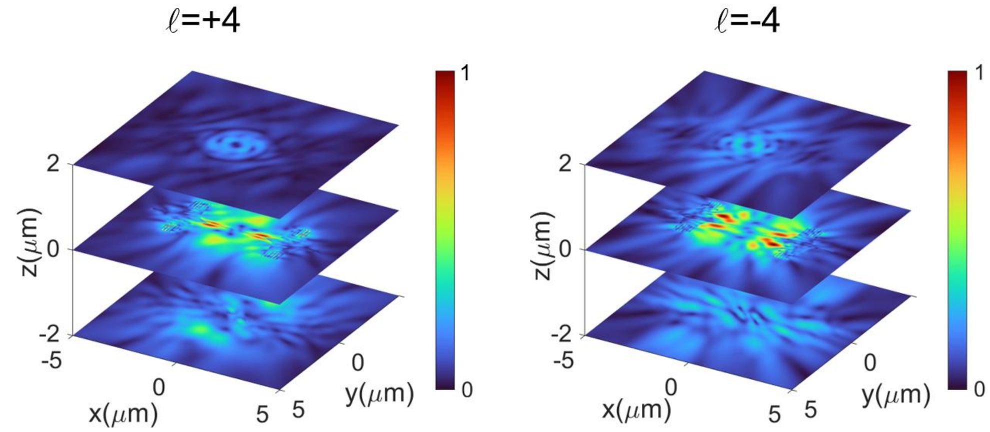

Fig. S6. Simulated intensity distributions of vortex beams with topological charge $\ell = \pm 4$ for the D-Cys nanoassembly. The cross-section at z=0 represents the middle of nanoassembly; *z*=2 and *z*=-2 correspond to the reflection and transmission planes, respectively.

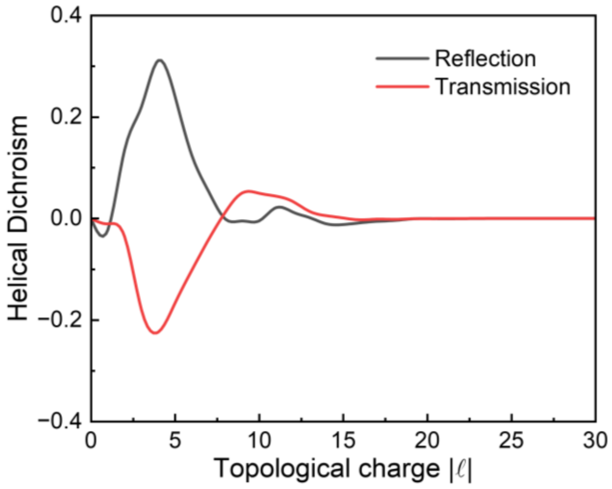

Fig. S7. Simulated chiroptical HD response of D-Cys nanoassemblies in transmission and reflection modes. The calculation formula for the HD signal in transmission mode is: $HD = 2 * \frac{T^{+} - T^{-}}{T^{+}_{max} + T^{-}_{max}}$, where T^{+} and T^{-} represent the transmitted intensities for vortex light with positive and negative topological charges, respectively, and T^{+}_{max} and T^{-}_{max} denote their maximum values.

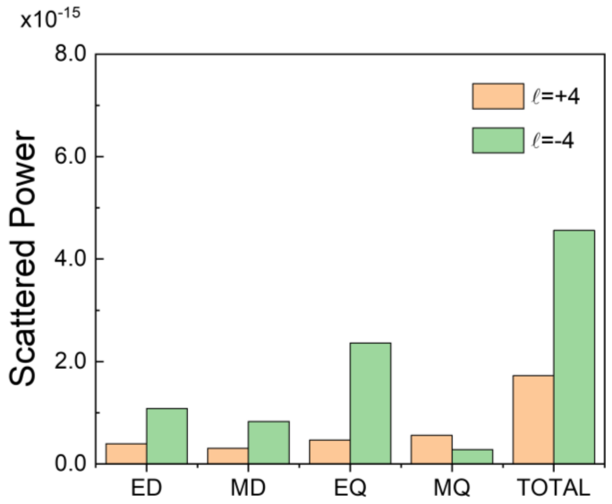

Fig. S8. Multipolar decomposition analysis of D-Cystine nanoassemblies. Contributions of the electric dipole (ED), magnetic dipole (MD), electric quadrupole (EQ), magnetic quadrupole (MQ), and total scattered power to the electromagnetic field response under incident vortex light with OAM states l = +4 and l = −4.

Supplementary Note 6: Influence of structural geometric parameters on HD signals

To further elucidate the chiroptical properties of L/D-Cys structures, we simulated the HD responses of chiral microstructures under different geometric parameters: the number of slices, rotation angle α, and thickness t (Fig. S9). Analysis of the simulation results in conjunction with the experimental data (dashed line) reveal that the closest match is achieved with a geometry comprising seven layers, a 5° angle of twist, and a layer thickness of 50 nm. In the synthesis process, maintaining a constant amino acid concentration, a decrease in cadmium chloride concentration result in an increase in the size of the resulting chiral microstructures (Table S1)[8]. The observed increase in the peak position of the chiroptical HD signal (Fig. S10) further supports this finding and aligns with previously reported results[10].

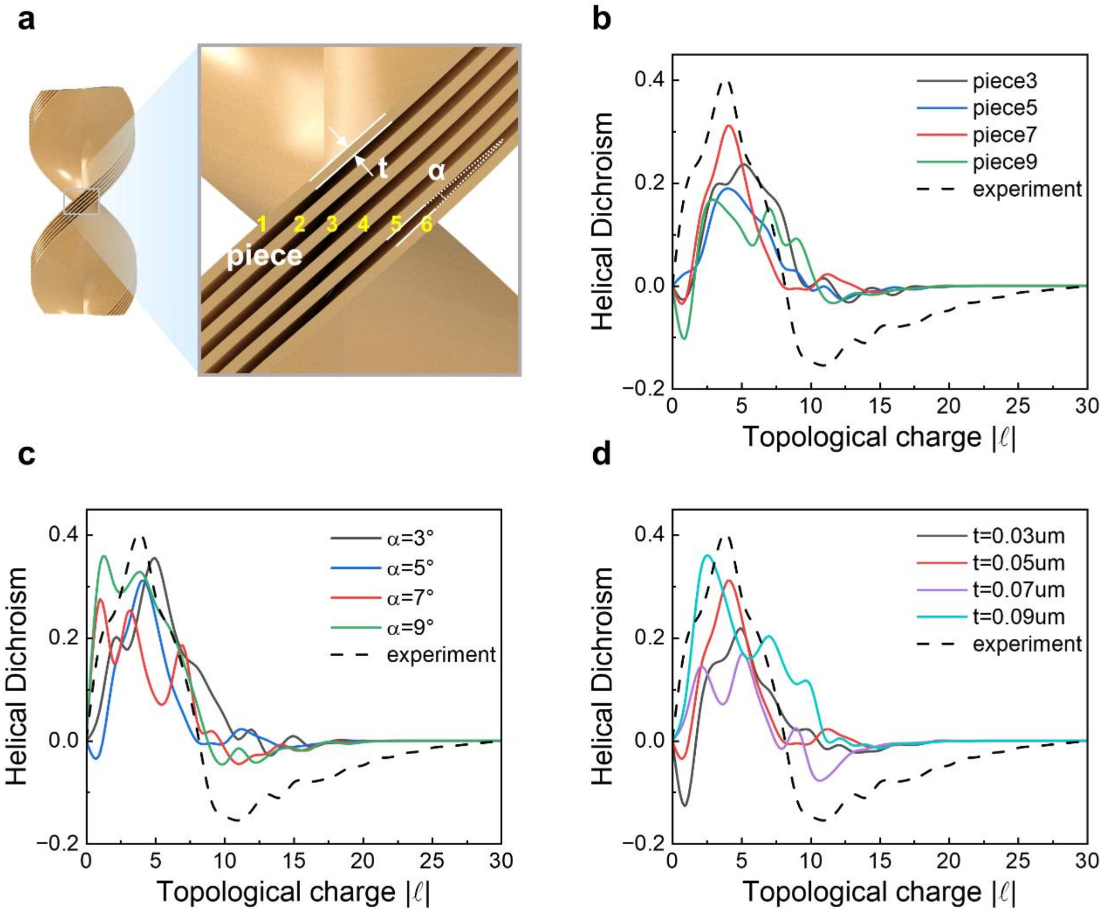

Fig. S9. Simulated HD signal by varying the number of slices, rotation angle α, and thickness t. a Schematic diagram of the different structural parameters: the number of

slices, rotation angle α, and thickness t. **b-d** The influence of the three parameters on the chiroptical HD signal for D-cys structures. The dashed line represents the experimental data, as shown in Fig. 3f.

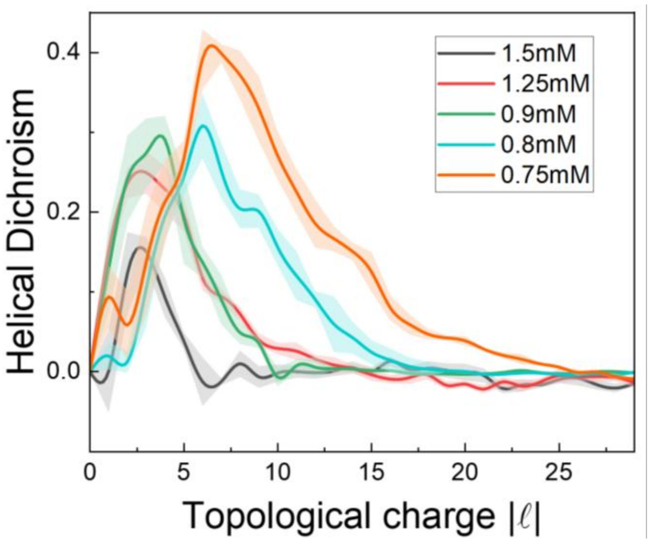

Fig. S10. Experimental HD signal measurements by varying the concentration of cadmium chloride solution while holding the concentration of D-cystine solution constant at 1 mM. As the concentration of cadmium chloride solution decreases, the size of the synthesized structures increases, and the topological charge corresponding to the peak in the chiroptical HD spectra gradually increases. Solid lines show the mean value and the shading indicates the standard deviation of multiple measurements.

Table S1: Structural dimensions of nanoassemblies as a function of cadmium chloride solution concentration.

D-cystine	$CdCl_2$	Length/μm	Width
1 mM	1.5 mM	5.6	2.7
1 mM	1.25 mM	7.2	3.4
1 mM	0.9 mM	7.6	3.6
1 mM	0.8 mM	11.0	5.1
1 mM	0.75 mM	13.3	5.6

Supplementary Note 7: Influence of beam-structure spatial alignment on HD signals

It is well recognized that the HD signal exhibits a high sensitivity to beam-center alignment. To address this, we have performed detailed simulations to investigate the variation of the HD signal as the beam center is offset along the *x* or *y* direction (Fig. S11). Our findings reveal that when the beam offset exceeds 0.5 μm in either the *x* or *y* direction, there is a noticeable reduction in the peak intensity of the signal. Furthermore, the topological charge corresponding to the peak undergoes a significant shift under these conditions. These results provide valuable insights into the robustness of the HD signal with respect to spatial alignment deviations.

Additionally, we conducted simulations to investigate how the orientation of the nanoassembly relative to the laser beam influences the HD signal (Fig. S12). Our simulations demonstrate that when the nanocrystal is rotated along the *x*-axis by an angle $\alpha<10°$, the HD signal peak is only minimally affected, exhibiting a slight decrease of intensity. However, when α increases to 20°, the peak intensity decreases to approximately half of its original value, accompanied by noticeable changes in the curve shape. Similarly, for rotations along the *z*-axis at an angle $\gamma<40°$, the HD signal peak exhibits a modest reduction. When γ increases to 80°, the peak intensity is reduced to half of its original value.

These findings underscore the sensitivity of the HD signal to the orientation of the nanoassembly. All simulations presented in Supplementary Note 6 are conducted under the operating wavelength of 800 nm.

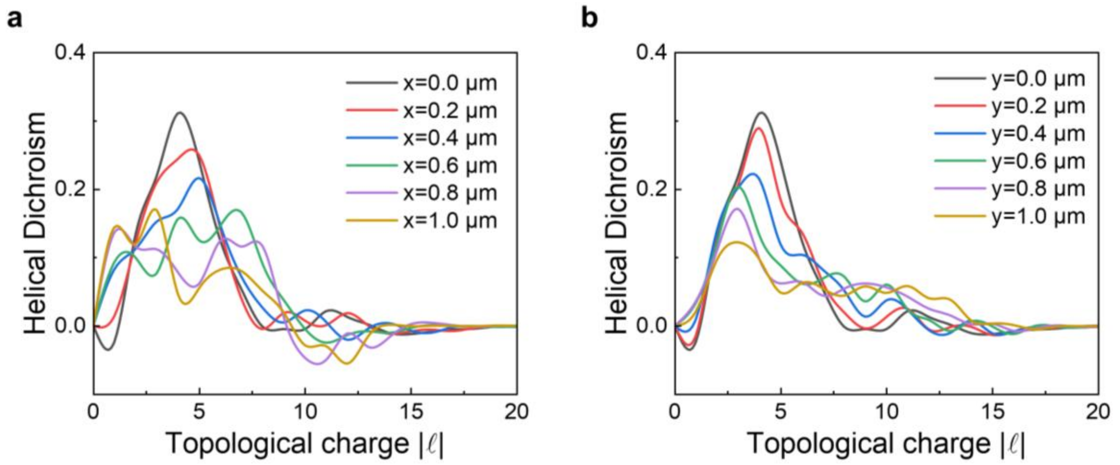

Fig. S11. Simulated chiroptical HD signal as a function of the beam center offset relative to the nanoassembly in the *x* and *y* directions. a-b Simulated HD signal obtained by varying the beam center offset along the *x* and *y* directions.

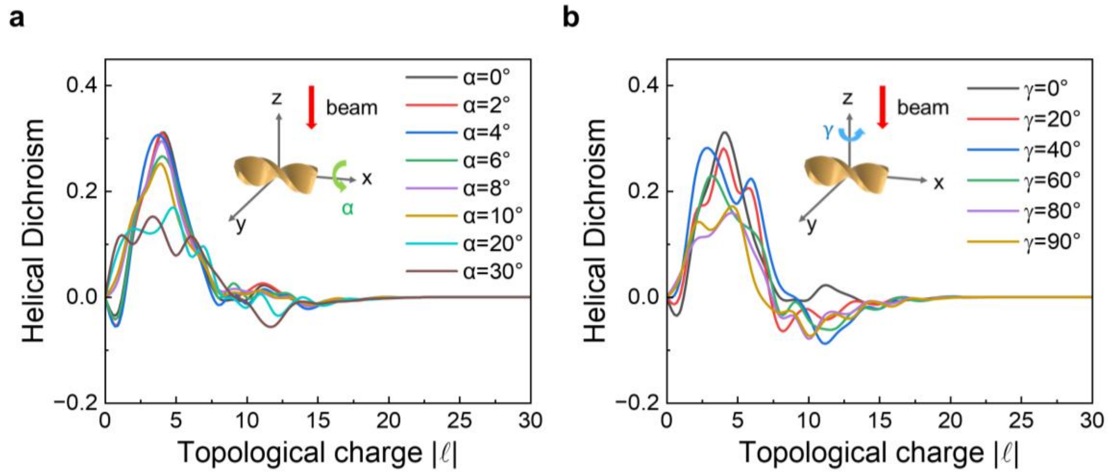

Fig. S12. Simulated chiroptical HD signal as a function of the nanoassembly orientation relative to the laser beam under *x*-polarized vortex light illumination. a-b Simulated HD signal obtained by varying the angle of rotation relative to the *x* (*z*) axis. (Inset) Schematic diagram of the different angels.

Supplementary Note 8: Circular dichroism from a single nanoassembly under tight focusing condition

We experimentally measured the circular dichroism (CD) from a single chiral nanoassembly under tightly focused conditions (Fig. S13). Across the wavelength range of 500-750 nm, the measured asymmetry factor of the CD signal is below 0.1, suggesting a relatively weak interaction between the circularly polarized light and the nanoassembly.

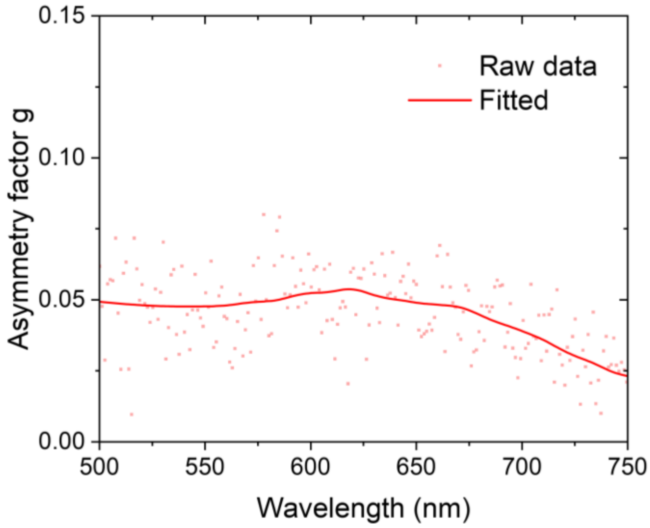

Fig. S13. Experimental CD signal on a single chiral nanoassembly. Data points represent the raw data, and the solid line is the fitted curve.

Supplementary Note 9: HD signal in photoluminescence emission

To better explain the origin of HD_{PL}, we conduct additional measurements of HD for the same nanoassembly in both the fundamental wavelength and photoluminescence emission, and discovered that the topological charge corresponding to the peak for HD is approximately the same for reflection and emission (Fig. S14). This is because the stronger reflection corresponds to a specific handedness of OAM light that interacts more strongly with the nanoassembly, which results in stronger photoluminescence emission. The emission characteristics are also investigated. By placing a linear polarizer in front of the spectrometer and varying its rotation angle, the results indicate that the photoluminescence is predominantly linearly polarized, with the polarization direction aligned along the long axis of the nanoassembly (Fig. S15).

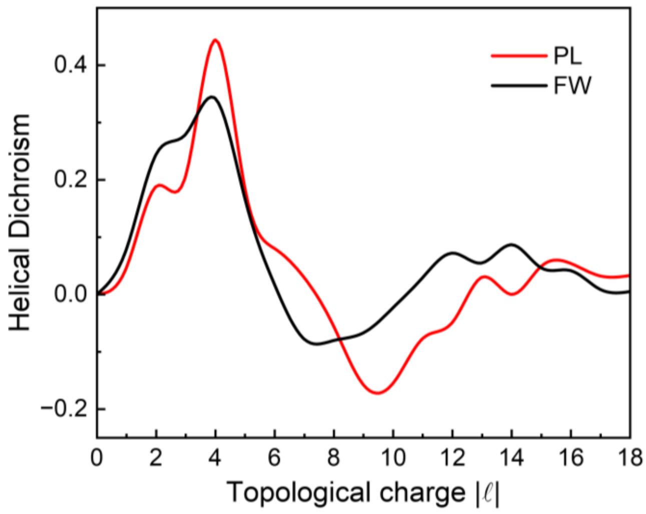

Fig. S14. Experimental HD signal from the same nanoassembly in the fundamental wavelength and photoluminescence emission. FW: fundamental wavelength; PL: photoluminescence.

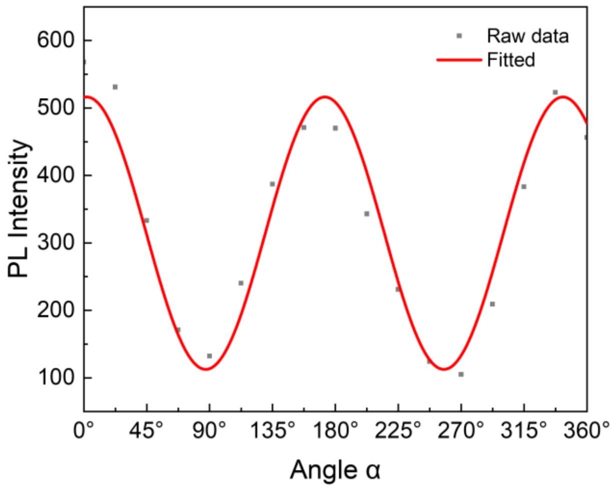

Fig. S15. Dependence of PL intensity on the rotation angle of the linear polarizer.

Data points represent the raw data, and the solid line is the fitted curve.

Table S2: Comparison of relevant reports for chiroptical molecular sensing.

Type	Geometry	Molecules	Method	Optical characterization	Wavelength	g factor	Reference
Metasurface	Gammadia	Tryptophan and the β-sheet proteins	Lithography	CD	~650nm	0.1	*Nat. Nanotechnol.* **5**, 783–787 (2010)
	Acute angle	1,2-Propanediol	Lithography	CD	~1000nm	<0.1	*Nat. Commun.* **8**, 14180 (2017)
Direct measurement	Chiral molecules	cysteine	/	CD	215nm	0.005	*Nature* **556**, 360–365 (2018)
	Chiral molecules	Fenchone	Nonlinear effects	HD	800nm	0.05	*Nat. Photon.* **17**, 82–88 (2023)
	Chiral molecules	$[Fe(4,4'\text{-diMebpy})_3]^{2+}$	Hard X-ray	HD	~0.17nm	0.05	*Nat. Photon.* **16**, 570–574 (2022)
Chemical synthesis	Nanoparticles (Co3O4)	Cysteine	Chemical synthesis	CD	~550nm	0.02	*Science* **359**, 309–314 (2018)
	Nanorods (Au)	Bovine serum albumin (BSA)	Self-assembly	CDS	~790nm	0.3	*Science* **365**, 1475–1478 (2019)
	Micelle	1,1′-binaphthyl-2,2′-diamine (BBINAMINE)	Micelle-directed growth	CD	~1000nm	0.2	*Science* **368**, 1472–1477 (2020)
	Nanoparticle-on-mirror (Au)	Oligoamide	Drop-cast	CDS	~650nm	0.2	*Nat. Commun.* ***15***,*2* (2024)
	Helices (CdTe)	Cysteine	Self-assembly	CD	~900nm	0.01	*Sci. Adv.* **3**, e1601159 (2017)
	Nanoparticles (Au)	Cysteine， glutathione	Chemical synthesis	CD	622nm	0.2	*Nature* **556**, 360–365 (2018)
	Nanoparticles (Au)	Cysteine	Chemical synthesis + Circular polarized light	CD	~600nm	0.4	*Nature* **612**, 470–476 (2022)
	Nanohelices (CdTe)	Cysteine	Self-assembly	THMS (Third-harmonic Mie scattering)	365nm	0.2	*Nat. Photon.* **16**, 126–133 (2022)
	**Nanoassemblies**	**Cystine**	**Self-assembly**	**HD**	**800nm**	**0.53 (FW) 1.18 (PL)**	**This work**

FW: fundamental wavelength; PL: photoluminescence.

References

1. Leutenegger, M. & Lob, E. S. I. Fast focus field calculations. *Opt.Express* **14**, 11277-11291 (2006).
2. Lin, J., Rodríguez-Herrera, O. G., Kenny, F., Lara, D. & Dainty, J. C. Fast vectorial calculation of the volumetric focused field distribution by using a three-dimensional Fourier transform. *Opt. Express* **20**, 1060 (2012).
3. Hu, Y. *et al.* Efficient full-path optical calculation of scalar and vector diffraction using the Bluestein method. *Light Sci. Appl.* **9**, 119 (2020).
4. Allen, L., Beijersbergen, M. W., Spreeuw, R. J. C. & Woerdman, J. P. Orbital angular momentum of light and the transformation of Laguerre-Gaussian laser modes. *Phys. Rev. A* **45**, 8185–8189 (1992).
5. Babiker, M., Bennett, C. R., Andrews, D. L. & Dávila Romero, L. C. Orbital Angular Momentum Exchange in the Interaction of Twisted Light with Molecules. *Phys. Rev. Lett.* **89**, 143601 (2002).
6. Bliokh, K. Y., Dressel, J. & Nori, F. Conservation of the spin and orbital angular momenta in electromagnetism. *New J. Phys.* **16**, 093037 (2014).
7. Bliokh, K. Y., Rodríguez-Fortuño, F. J., Nori, F. & Zayats, A. V. Spin–orbit interactions of light. *Nat. Photon.* **9**, 796–808 (2015).
8. Kumar, P. *et al.* Photonically active bowtie nanoassemblies with chirality continuum. *Nature* **615**, 418–424 (2023).
9. Alaee, R., Rockstuhl, C., & Fernandez-Corbaton, I. An electromagnetic multipole expansion beyond the long-wavelength approximation. *Optics Communications* **407**, 17-21 (2018).
10. Ni J. *et al.* Gigantic vortical differential scattering as a monochromatic probe for multiscale chiral structures. *Proc. Natl Acad. Sci. USA* **118**, e2020055118 (2021).
